# The common regulatory pathway of COVID-19 and multiple inflammatory diseases and the molecular mechanism of cepharanthine in the treatment of COVID-19

**DOI:** 10.3389/fphar.2022.960267

**Published:** 2022-07-22

**Authors:** Ping Jiang, Jingyao Ye, Menglong Jia, Xiaopeng Li, Shujun Wei, Nianhu Li

**Affiliations:** ^1^ Shanghai University of Traditional Chinese Medicine, Shanghai, China; ^2^ Guanghua Clinical Medical College, Shanghai University of Traditional Chinese Medicine, Shanghai, China; ^3^ Shandong University of Traditional Chinese Medicine, Jinan, China; ^4^ Weifang Hospital of Traditional Chinese Medicine, Weifang, China; ^5^ Rizhao Hospital of Traditional Chinese Medicine, Rizhao, China; ^6^ Affiliated Hospital of Shandong University of Traditional Chinese Medicine, Jinan, China

**Keywords:** COVID-19, cepharanthine, network pharmacology, new use of old drugs, homotherapy for heteropathy

## Abstract

**Background:** Similar pathogenesis makes Corona Virus Disease 2019 (COVID-19) associated with rheumatoid arthritis (RA), ankylosing spondylitis (AS) and gouty arthritis (GA), and it is possible to introduce common drugs for the treatment of RA, AS and GA into the treatment of COVID-19. That is, “homotherapy for heteropathy”, especially cytokine inhibitors. But little is known about the specific link between the diseases. In addition, “new use of old drugs” is an important short-term strategy for the treatment of COVID-19. Cepharanthine (CEP), a monomer component of traditional Chinese medicine (TCM), is mainly used in the treatment of leukopenia and has recently been proved to have a good therapeutic effect on COVID-19, but its specific molecular mechanism has not been clearly explained. The purpose of this work is to explore the common targets and signaling pathways among COVID-19, RA, AS, and GA by means of network pharmacology (NP), and to infer the potential mechanism of CEP in the treatment of COVID-19.

**Methods:** Firstly, SwissTargetPrediction was used to predict the targets of CEP, and the pathogenic targets of COVID-19, RA, AS and GA were searched in GeneCards, OMIM, TTD, PharmGKB database and literature, respectively. Then, the protein interaction network of CEP and COVID-19 cross targets and the common targets of COVID-19, RA, AS and GA was constructed. Cytosscape 3.7.2 software was used to construct CEP-common targets-signaling pathways-COVID-19 network, module function analysis, gene ontology (GO) and kyoto encyclopedia of genes and genomes (KEGG). Finally, the molecular docking of hub targets and CEP was carried out by AutoDock software.

**Results:** The results showed that the common targets of the four diseases were tumor necrosis factor (TNF), interleukin (IL)-6 and IL-1β, and involved Coronavirus disease, IL-17 signaling pathway and TNF signaling pathway. CEP has a good binding force with AKT Serine/Threonine Kinase 1 (AKT1), phosphatidylinositol 3-kinase (PIK3) CA, PIK3CD and Angiotensin-converting enzyme 2 (ACE2), and plays a role in the treatment of COVID-19 by regulating PI3K-Akt signaling pathway, Relaxin signaling pathway, VEGF signaling pathway and HIF-1 signaling pathway.

**Conclusion:** Therefore, this study not only confirmed the potential mechanism of CEP in the treatment of COVID-19 at the molecular level, but also found that TNF and IL-17 inhibitors, which are commonly used in the treatment of RA, AS and GA, may also affect the treatment of COVID-19, which provides new clues and theoretical basis for the rapid discovery of effective therapeutic drugs for COVID-19.

## Introduction

“COVID-19” named “Corona Virus Disease 2019” by the World Health Organization (World Health Organization, 2020). Since December 2019, COVID-19 has been prevalent all over the world and has become one of the main causes of death in the world, posing a serious threat to human life and health. It is an infectious disease caused by severe acute respiratory syndrome coronavirus 2 (SARS-CoV-2) and this virus is highly contagious and spreads quickly (Chang et al., 2020; Liu Y.-C. et al., 2020). At present, although the vaccine and drug research for COVID-19 has made continuous progress, it is still a challenging process to effectively manage the development of new drugs for COVID-19. Therefore, the strategy of “new use of old drugs” is put forward to encourage the re-exploration of the use of existing drugs. The literature related to COVID-19 reported that ACE2 is the surface receptor of SARS-CoV-2 prickle glycoprotein and expressed in alveolar epithelial cells, which can promote SARS-CoV-2 to enter and infect host cells (Hoffmann et al., 2020). The interaction between ACE2 and SARS-CoV-2 down-regulates the expression of ACE2 and activates AngⅡ receptor, resulting in increased pulmonary vascular permeability and lung injury (Zhao et al., 2020). Other studies have found that ACE insertion-deletion (I/D) gene polymorphism is also associated with RA and AS. In the study of DD, ID and II alleles, the frequency of D allele in RA patients was higher than that in healthy controls, while DD gene may increase the susceptibility to RA and cause sacral and eye involvement in AS patients (Inanır et al., 2012; Ahmed et al., 2013; Elshazli et al., 2022). Therefore, ACE2 may be related to the pathological process and inflammatory reaction of RA and AS. In addition, COVID-19 is associated with a high inflammatory process. SARS-CoV-2 infection can induce the human body to produce a large number of cytokines, including IL-6, TNF-α and IL-1β, which gather inflammatory cells to the infected site, thereby destroying tissues and organs (Lai et al., 2020). A variety of cytokines and chemokines play a role in the pathogenesis of RA, AS and GA by regulating inflammation, autoimmunity and joint destruction, including IL-1β, IL-6, IL-17, TNF-α, and CXCL8. In arthritis, RA, AS and GA are three common arthritis diseases, which have a high prevalence rate in the population, and they are closely related and can be combined, so they have some similar pathogenesis and treatment. The clinical research on the three diseases is very rich, and the related therapeutic drugs are also being studied and developed continuously. Interestingly, through the analysis of recent related studies, it is not difficult to find that there are some similarities between COVID-19 and RA, AS, GA in pathogenesis, clinical manifestations and drug treatment. Accordingly, the similarity of pathogenesis introduces drugs commonly used in the treatment of RA, AS and GA into COVID-19. At present, a number of clinical studies have been carried out to down-regulate the levels of inflammatory factors in patients with COVID-19, including monoclonal antibodies against IL-6 and IL-1β, and inhibitors of JAK-STAT signaling pathway. For example, IL-6 receptor blocker tocilizumab is mainly used in RA, and it has been proved to be effective in relieving the symptoms of COVID-19 (Favalli et al., 2020). Tofacitinib is a kind of Janus Kinase (JAK) inhibitor, which can block the signal transduction of a variety of inflammatory cytokines. It has a good effect on the treatment of RA, AS and other inflammation-related diseases (Burmester et al., 2013). A tofacitinib study in Brazil of hospitalized patients with COVID-19 showed (Guimarães et al., 2021) that the tofacitinib group (18.1%) had a lower cumulative incidence of death or respiratory failure on day 28 than the placebo group (29.0%). In addition, TNF and IL-17 inhibitors, which have been used clinically for many years, have been widely used in the treatment of RA, AS, GA and other diseases. If new indications of TNF and IL-17 inhibitors can be found, the time period of drug development will be greatly shortened and the success rate of drug discovery for COVID-19 will be improved. In addition, TCM has accumulated more experience in the treatment of epidemic diseases. The treatment characteristics of multi-components, multi-targets and multi-pathways of TCM show the advantages of prevention and treatment of COVID-19, and can complement with western medicine to improve the therapeutic effect. CEP, a monomer component of TCM, is a natural alkaloid extracted from Stephania japonica, which has the effects of anti-inflammation, antioxidation, immune regulation and antivirus (Wang and Yang, 2020). It is used in the treatment of leukopenia. As early as 2020, Tong et al. (Fan et al., 2020) has reported the research results of anti-COVID-19 drugs, and found that CEP is a potential drug for the treatment of SARS-CoV-2 infection. The RNA sequencing revealed the response and antiviral activity of cells to viruses, and confirmed that CEP could reverse most of the dysfunctional genes and pathways in virus-infected cells through HSF1-mediated heat shock reaction, which provided evidence for CEP as a promising therapeutic drug (Li et al., 2021). Since then, a number of research teams in the world have published articles on CEP anti-SARS-CoV-2 in international journals, which shows that CEP has a good anti-novel coronavirus activity (Drayman et al., 2021; Ohashi et al., 2021). Therefore, as an antiviral drug, it has important value in anti-SARS-CoV-2 (Rogosnitzky et al., 2020).

Network Pharmacology (NP) uses database information of drugs, compounds, genes and diseases to construct drug targets, disease targets and signaling pathways interaction network, in order to reveal the complex mechanism of multi-component and multi-target characteristics of TCM, and provides a basis for the transition of TCM from empirical medicine to evidence-based medicine (Hopkins, 2008; Li and Zhang, 2013).

**FIGURE 1:**
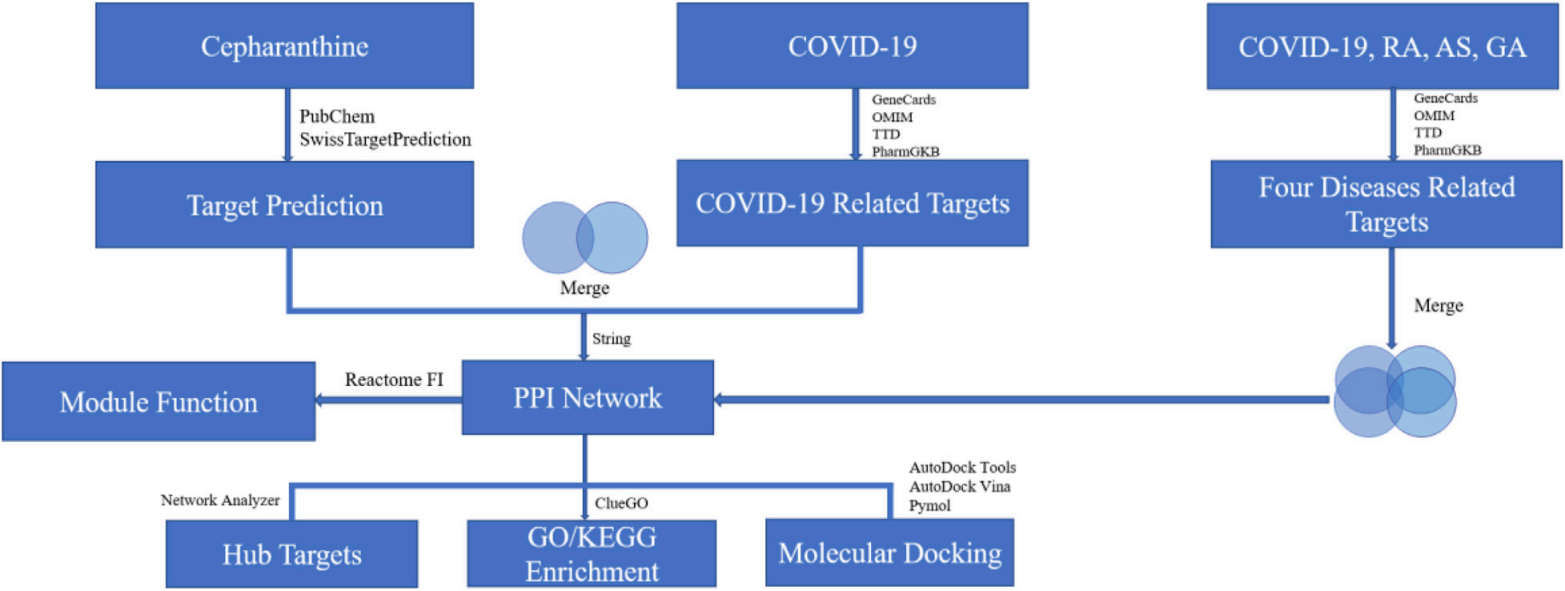
Workflow diagram of the NP.

Based on the above research background, this study will explore the common targets and signaling pathways among COVID-19, RA, AS and GA diseases and the potential mechanism of CEP in COVID-19 through the methods of NP (Figure 1), so as to provide a theoretical basis for the exploration of the treatment strategy of “homotherapy for heteropathy” and “new use of old drugs”.

## Methods

### Acquisition target genes of active compound

The Canonical SMILES structural formula of CEP is obtained by using PubChem (https://pubchem.ncbi.nlm.nih.gov/) website, and then the structural formula is imported into SwissTargetPrediction (http://www.swisstargetprediction.ch/) website (Daina et al., 2019) to predict and obtain the action targets of CEP, and consult the literature to supplement targets.

### Disease target genes

The pathogenic genes related to COVID-19 were obtained by using GeneCards (https://www.genecards.org/) (Stelzer et al., 2016), OMIM (https://omim.org/) (Amberger et al., 2015), TTD (https://db.idrblab.org/ttd/) (Wang et al., 2020), PharmGKB (https://www.pharmgkb.org/) (Whirl-Carrillo et al., 2021) database. The key words “COVID-2019” and “Corona Virus Disease 2019” were utilized to screen the COVID-19-associated targets. We established a COVID-19–related gene set by taking a union of the search results. RA, AS and GA related genes were obtained respectively by the same method, and then a data set for COVID-19, RA, AS and GA target genes was established.

### Common target genes of disease-disease and disease-compound

By using the online tool of Venny 2.1.0, the targets of four diseases in the combination of multiple diseases (COVID-19, RA, AS, GA) were intersected, and the targets of active compound were cross-located with the targets of COVID-19. Finally, the common targets of the four diseases and the potential targets of CEP in the treatment of COVID-19 were obtained.

### Constructing a protein-protein interactions network of common targets and screening hub targets

A network of interactions between common targets is constructed and analyzed through the STRING (https://string-db.org) database (Szklarczyk et al., 2021), which provides information about predicting and experimenting protein-protein interactions. The results of interaction analysis are imported into Cytoscape 3.7.2 software and the CytoHubba plug-in is used to analyze the connectivity (Degree) of the data in protein-protein interactions (PPI) network to select the hub targets of ranking TOP10.

### GO and KEGG enrichment analysis

GO and KEGG enrichment analysis of common targets using Cytoscape 3.7.2 software ClueGO plug-in. The minimum gene overlap was set to three and the *p* value was less than 0.01. GO enrichment analysis includes three modules: biological process, molecular function and cellular composition. KEGG provides pathways’ functional annotation and enrichment analysis of a given gene. Finally, we get the relevant GO and KEGG data, and sort by *p* value, showing the results of ranking TOP10 or TOP20.

### Module function analysis

Module function analysis is based on a complex algorithm for clustering objects with similar attributes. The Analyze Module Functions of Reactome FI (Croft et al., 2011) plug-in of Cytoscape3.7.2 software was used to cluster and analyze the common targets of multi-disease combinations (COVID-19, RA, AS, GA). The results were visualized and output in graphics.

### Molecular docking

The RCSB PDB (https://www1.rcsb.org/) database is used to retrieve and download the PDB ID of the potential target protein, while the MOL2 structure format of the active compound is downloaded in the TCMSP (http://tcmspw.com/tcmsp.php) (Ru et al., 2014). Then CEP and the hub targets were analyzed by AutoDock Tools software, and CEP ligand and target protein were converted into “PDBQT” format, and the box of appropriate size and position was set. Finally, the molecular docking was carried out by using AutoDock Vina software, the molecular binding activity between target protein and CEP was calculated, and the results of strong binding activity were visually displayed by Pymol software.

## Results

### Analysis of interaction targets and signaling pathways of COVID-19, RA, AS, and GA

**FIGURE 2:**
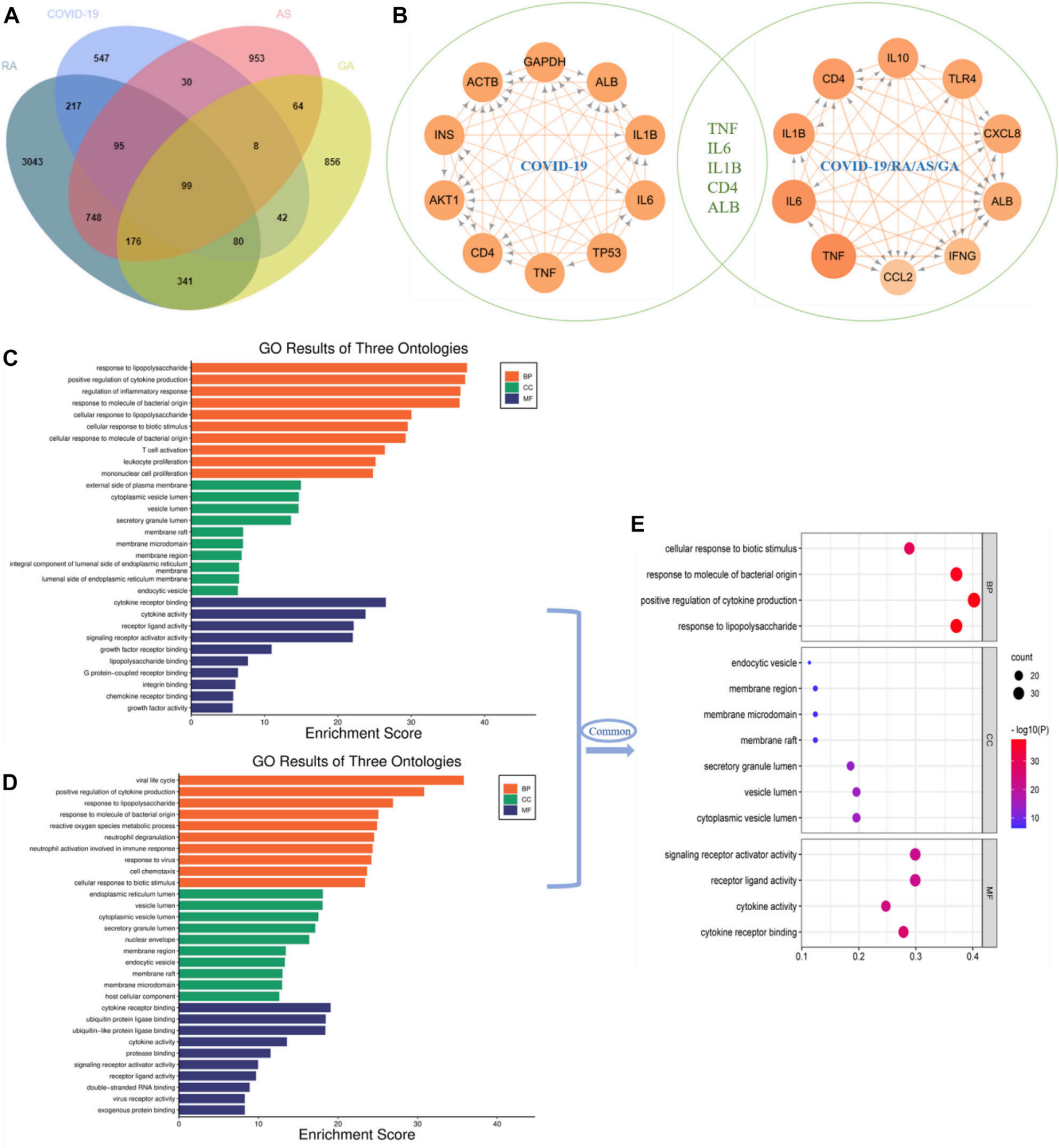
The common hub targets and GO process of single disease (COVID-19) and multiple disease combinations (COVID-19, RA, AS, GA). **(A)** Multi-disease combination (COVID-19, RA, AS, GA) 99 common targets. **(B)** Single disease (COVID-19) targets ranked TOP10 targets (left), multi-disease combination (COVID-19, RA, AS, GA) common targets ranked TOP10 targets (right), the intersection of the two, five common targets were obtained: TNF, IL-6, IL-1β, CD4 and ALB, these five targets not only play a central role in the pathogenesis and progression of single disease (COVID-19), but also are potential targets for the treatment of RA, AS and GA. **(C)** The common GO process involved in multiple disease combinations (COVID-19, RA, AS, GA). **(D)** The GO process involved in single disease (COVID-19). **(E)** The common GO process obtained by the intersection analysis of single disease (COVID-19) and multiple disease combinations (COVID-19, RA, AS, GA), and mainly involves response to lipopolysaccharide, positive regulation of cytokine production, cytokine receptor binding.

**FIGURE 3:**
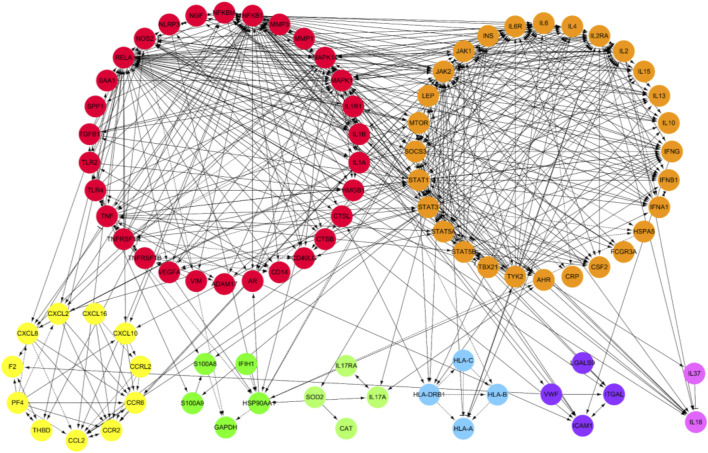
Module function analysis of common targets of multiple disease combinations (COVID-19, RA, AS, GA). After the functional analysis of the module, the 99 targets can be aggregated into eight modules, and the circle of each color represents one module, which is mainly related to immune response, inflammatory progression, bacteria and virus expression and other functions. And there is a close relationship between the enriched targets on each module, and also has a certain correlation with the targets on other modules.

**FIGURE 4:**
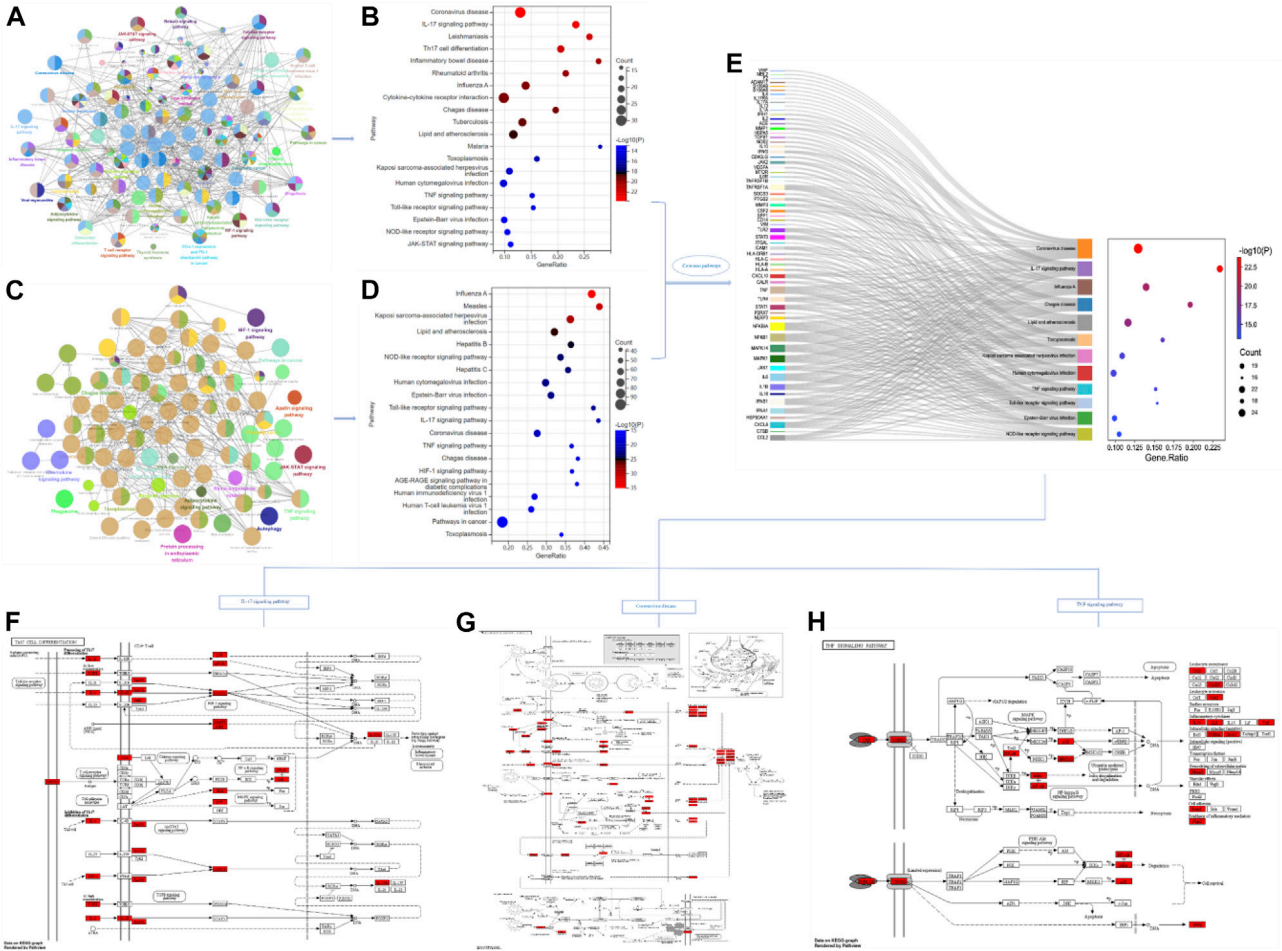
Common signaling pathways of single disease (COVID-19) and multiple disease combinations (COVID-19, RA, AS, GA). **(A,B)** The common signaling pathways involved in multiple disease combinations (COVID-19, RA, AS, GA) and the pathways that rank TOP20 among them. **(C,D)** The signaling pathways regulated by single disease (COVID-19) and the pathways of ranking TOP20. **(E)** Based on the intersection analysis of TOP20 pathways in single disease (COVID-19) and multiple disease combinations (COVID-19, RA, AS, GA), 12 common regulated signaling pathways were obtained, including Coronavirus disease, TNF signaling pathway, IL-17 signaling pathway, and showed the enriched targets on each pathway. **(F–H)** Specific signaling transduction pathways of Coronavirus disease, TNF signaling pathway and IL-17 signaling pathway.

Through database search, after removing duplication and combining the search results, a total of 1,118 genes related to single disease (COVID-19) were obtained. After calculation and analysis, the hub targets of TOP10 were CD4, AKT1, INS, ACTB, GAPDH, ALB, IL-1β, IL-6, TP53 and TNF (Figures 2A,B). In the same way, the action targets of RA, AS and GA were obtained, and combined with 1,118 targets of COVID-19, 99 common targets of the four diseases were found (Figure 2A). The hub targets of TOP10 were TNF, IL-6, CD4, IL-1β, IL-10, TLR4, CXCL8, ALB, IFNG, CCL2 (Figure 2B). Compared with the hub targets of single disease (COVID-19), it is found that TNF, IL-6, IL-1β, CD4 and Albumin (ALB) are common (Figure 2B), indicating that these five targets not only play an important role in the common pathogenesis of COVID-19, RA, AS and GA, but also play a core role in the pathogenesis of single disease (COVID-19) and are potential targets for the treatment of COVID-19. After that, GO enrichment analysis was performed. The results showed that the common GO processes of COVID-19, RA, AS and GA mainly includes response to lipopolysaccharide, regulation of inflammatory response and cytokine receptor binding (Figure 2C) and the pathogenesis of COVID-19 was mainly involved in the processes of positive regulation of cytokine production, response to lipopolysaccharide and cytokine receptor binding (Figure 2D). Based on the above results, the common biological processes involved in COVID-19, RA, AS and GA were combined with the results of single disease (COVID-19) analysis. It was found that they jointly regulate the biological processes such as response to lipopolysaccharide, positive regulation of cytokine production and cytokine receptor binding (Figure 2E). Then, 99 common targets were analyzed by module function. The results showed that 99 targets could be aggregated into eight modules, such as Coronavirus disease-COVID-19, Toll-like Receptor Cascades, Toll-like receptor signaling pathway, Rheumatoid arthritis, NF-kappa B signaling pathway, MAPK signaling pathway, TNF signaling pathway, IL-17 signaling pathway, Th17 cell differentiation, which were mainly involved in immune response, inflammatory progression and bacterial and viral expression (Figure 3). Finally, KEGG analysis is carried out. The results showed that the common KEGG pathways of COVID-19, RA, AS and GA mainly includes Coronavirus disease, IL-17 signaling pathway, Th17 cell differentiation, TNF signaling pathway and Toll-like receptor signaling pathway (Figures 4A,B). The pathogenesis of COVID-19 was mainly involved and regulation of multiple pathways such as NOD-like receptor signaling pathway, Toll-like receptor signaling pathway, IL-17 signaling pathway, Coronavirus disease, TNF signaling pathway and HIF-1 signaling pathway (Figures 4C,D). Based on the above results, the common signaling pathways involved in COVID-19, RA, AS and GA were combined with the results of single disease (COVID-19) analysis. It was found that they jointly regulate the Coronavirus disease, TNF signaling pathway and IL-17 signaling pathway (Figure 4E; Table 1). The above results may indicate that these biological processes and signaling pathways are closely related to the pathogenesis of COVID-19, which is a potential way to regulate the progression of COVID-19 and implement the strategy of “new use of old drugs”.

**TABLE 1:** The analysis results of important signaling pathways.

Signaling pathways	Number of genes	Contained genes
Coronavirus disease	30	ACE/ADAM17/CCL2/CSF2/CXCL10/CXCL8/F2/IFIH1/IFNA1/IFNB1/IL1β/IL2/IL6/IL6R/JAK1/MAPK1/MAPK14/MBL2/MMP1/MMP3/NFKB1/NFKBIA/NLRP3/STAT1/STAT3/TLR2/TLR4/TNF/TNFRSF1A/VWF
TNF signaling pathway	17	CCL2/CSF2/CXCL10/ICAM1/IFNB1/IL1β/IL6/MAPK1/MAPK14/MMP3/NFKB1/NFKBIA/PTGS2/SOCS3/TNF/TNFRSF1A/TNFRSF1B
IL-17 signaling pathway	22	CCL2/CSF2/CXCL10/CXCL8/HSP90AA1/IFNG/IL13/IL17A/IL17RA/IL1β/IL4/IL6/MAPK1/MAPK14/MMP1/MMP3/NFKB1/NFKBIA/PTGS2/S100A8/S100A9/TNF
PI3K-Akt signaling pathway	11	EGFR/NOS3/JAK2/AKT1/PIK3CA/MTOR/PIK3CD/CDK4/CDK2/PIK3CG/BAD
Relaxin signaling pathway	8	EGFR/NOS3/MMP1/AKT1/PIK3CA/NOS2/PIK3CD/MAPK14
VEGF signaling pathway	6	NOS3/AKT1/PIK3CA/PIK3CD/MAPK14/BAD
HIF-1 signaling pathway	7	EGFR/NOS3/AKT1/PIK3CA/MTOR/NOS2/PIK3CD

### Potential mechanism of CEP in the treatment of COVID-19

**FIGURE 5:**
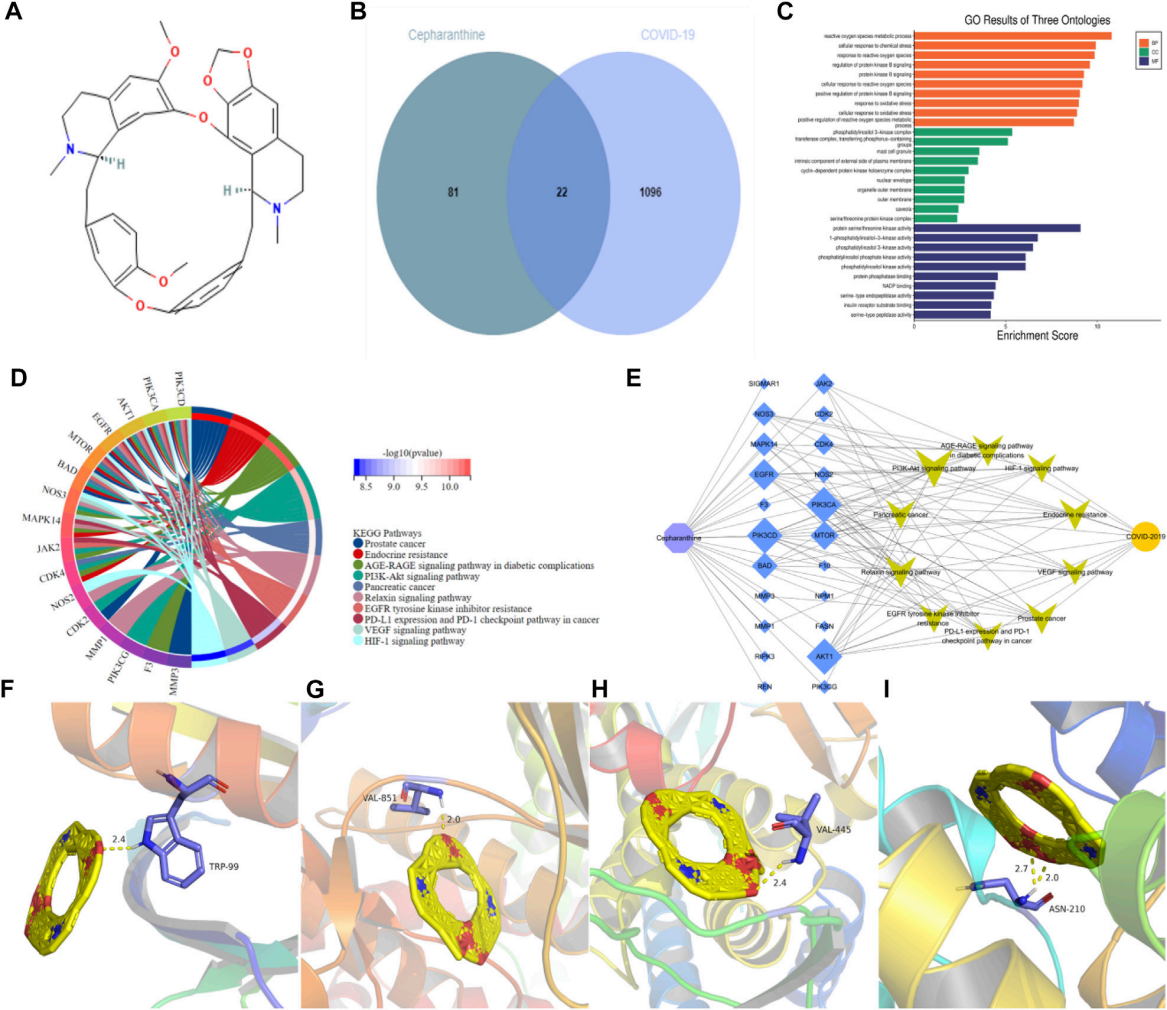
Potential molecular mechanism of CEP in the treatment of COVID-19. **(A)** The molecular structure of CEP is a double segment isovaline alkaloid with a molecular formula of C_37_H_38_N_2_O_6_. **(B)** Which is the common targets of CEP and COVID-19. **(C)** Results of GO analysis shows the process of ranking TOP10, which mainly involves cell proliferation, cell biological regulation and protein transcription and translation. **(D)** Results of KEGG analysis mainly regulate signaling pathways such as PI3K-Akt signaling pathway, Relaxin signaling pathway, VEGF signaling pathway and HIF-1 signaling pathway. **(E)** CEP-action targets-signaling pathways-COVID-19 association network, purple represents the TCM component CEP, blue represents the action targets, the larger the shape, the more important the target, yellow is the signaling pathways, orange represents the disease COVID-19, showing that CEP has the characteristics of multi-targets and multi-pathways therapy. **(F–I)** Molecular docking result of CEP and AKT1, PIK3CA, PIK3CD, ACE2.

CEP is a bisbenzyl isowaline alkaloid, its molecular structure is shown in the figure (Figure 5A). After intersecting with COVID-19-related targets, 22 common targets (Figure 5B) were obtained. These 22 targets may be potential targets of CEP in the treatment of COVID-19. Then 22 targets were analyzed by GO and KEGG, and sorted by *p* value to show the GO process and signaling pathways of TOP10. The results showed that cell proliferation, cell biological regulation, nucleic acid metabolism and protein transcription and translation were mainly involved in GO processes (Figure 5C), and CEP mainly played a role in the treatment of COVID-19 by regulating PI3K-Akt signaling pathway, Relaxin signaling pathway, VEGF signaling pathway and HIF-1 signaling pathway (Figure 5D; Table 1). Finally, the CEP-targets-signaling pathways-COVID-19 network diagram is drawn by Cytoscape3.7.2 software (Figure 5E), and the hub targets such as AKT1, PIK3CA and PIK3CD are selected according to the network connectivity (Degree). Because SARS-CoV-2 can only enter cells that can express ACE2 and bind to ACE2 through S-protein, and then invade the body to cause disease. ACE2 plays a very important role in the pathogenesis of COVID-19, subsequent molecular docking will increase the analysis between ACE2 and CEP. When CEP is docked with AKT1, PIK3CA, PIK3CD and ACE2, it is generally believed that the binding energy less than −4.25 kcal/mol indicates that there is a certain binding activity between the receptor and the ligand, the binding energy less than −5.0 kcal/mol indicates that the receptor and the ligand have a better binding activity, and the binding energy less than −7.0 kcal/mol indicates a strong binding activity between the receptor and the ligand. The results show that CEP has strong binding activity (Figures 5F–I; Table 2) to these four key proteins, indicating that CEP can effectively act on these targets and indirectly regulate the pathways enriched by the targets to play a corresponding therapeutic role.

**TABLE 2:** Molecular binding activity of CEP to target protein.

Test compounds	Target protein	PDB ID	Bind energy (kcal/mol)
Cepharanthine	AKT1	1UNQ	−9.72
	PIK3CA	5UBR	−11.56
	PIK3CD	5DXU	−11.77
	ACE2	2AJF	−12.44

## Discussion

Syndrome differentiation and treatment is the quintessence of TCM and the basic rule to guide clinical diagnosis and treatment of diseases. It is under the guidance of this principle that “homotherapy for heteropathy” comes into being. “homotherapy for heteropathy” refers to different diseases. When the disease location is the same, the etiology is the same, and the pathogenesis coincides with each other, the same method can be used to treat different diseases. As one of the most basic treatment principles of TCM, it has a very important guiding significance for improving clinical efficacy. At present, symptomatic treatment is mostly used for COVID-19, and there is no specific treatment drug, so “homotherapy for heteropathy” combined with “new use of old drugs” can be used as a potential research and development strategy in theory.

The common targets, module function, biological processes and signaling pathways of multi-disease combinations (COVID-19, RA, AS, GA) were analyzed by the method of NP. In the functional analysis of the module, it can be found that mainly involves Coronavirus disease-COVID-19 and Rheumatoid arthritis disease modules and Toll-like Receptor Cascades, Toll-like receptor signaling pathway, NF-kappa B signaling pathway, MAPK signaling pathway, TNF signaling pathway, IL-17 signaling pathway, Th17 cell differentiation signaling pathway modules (Figure 3). These modules were mainly involved in immune response, inflammatory progression and bacterial and viral expression. Like Toll-like receptor 4 (TLR4) in Toll-like receptor signaling pathway, it is an innate immune receptor on the cell surface that recognizes pathogen-associated molecular pattern (PAMP), including viral proteins, and triggers the production of type I interferon and pro-inflammatory cytokines to fight infection (de Kleijn and Pasterkamp, 2003). TLR4 may play an important role in the pathogenesis of SARS-CoV-2, its overactivation may lead to persistent or excessive innate immune response, and innate immune response is the key to identify and control infection by releasing cytokines and chemokines. It was found that the expression of TLR2 and MyD88 was related to the severity of COVID-19. In terms of mechanism, TLR2 and MyD88 are necessary for inflammatory response induced by β-coronavirus. TLR2 takes SARS-CoV-2 envelope protein as ligand and relies on TLR2 signal to induce the production of inflammatory cytokines during coronavirus infection. In addition, blocking TLR2 signal *in vivo* can protect the pathogenesis of SARS-CoV-2 infection (Zheng et al., 2021). In addition, the enrichment results showed that the pathogenic processes of RA, AS and GA were all related to the targets of TNF, IL-6, IL-1β and part of the Coronavirus disease signaling pathway (Figure 4G), and there were some common pathogenesis, targets and biological processes with COVID-19. Such as immune disorder, inflammatory storm and response to lipopolysaccharide. Among them, IL-6 targets can not only induce B lymphocytes to proliferate, differentiate and secrete corresponding antibodies, but also promote the proliferation and activation of T lymphocytes, thus participating in anti-infective immunity and autoimmunity (Choy et al., 2020; McConnell et al., 2021). IL-1β is a typical pro-inflammatory cytokine. Inflammatory signals can stimulate the secretion of IL-1β by activating inflammatory bodies. A small amount of IL-1β can produce an appropriate inflammatory response to activate specific immune response and play an immune surveillance role (Apte and Voronov, 2008). Moreover, it involves many regulatory pathways related to immunity and inflammation, such as IL-17 signaling pathway, TNF signaling pathway, Toll-like receptor signaling pathway, and indicates that the etiology and pathogenesis of the four diseases are partly the same, which is the premise of “homotherapy for heteropathy”. After independent analysis of COVID-19, it was found that the pathological process was mainly related to response to lipopolysaccharide process and NOD-like receptor signaling pathway, Toll-like receptor signaling pathway, IL-17 signaling pathway, TNF signaling pathway, HIF-1 signaling pathway and so on. It was confirmed that some of the signaling pathways regulated by multiple disease combinations (COVID-19, RA, AS, GA) could theoretically be used as therapeutic pathways for COVID-19, such as TNF signaling pathway and IL-17 signaling pathway are closely related to viral infection and inflammatory response (Figures 4F,H). Studies have confirmed that TNF is secreted by mononuclear macrophages after activation and transformed into TNF-α after maturation, while TNF-α can inhibit viral replication, block viral protein synthesis and virion production, and has an antiviral effect similar to that of interferon (IFN) (Wong and Goeddel, 1986; Spratte et al., 2018). IL-17A is also a pro-inflammatory cytokine, mainly produced by Th17 cells, but also by innate immune cells and other acquired immune cells (Iwakura et al., 2011). It has been reported that IL-17 is associated with a variety of inflammatory respiratory diseases. The autocrine effect of IL-17 can stimulate human bronchial epithelial and venous endothelial cells to produce chemokines, thus promoting the inflow of neutrophils and aggravating respiratory tract inflammation (Mebratu and Tesfaigzi, 2018; Wang et al., 2019; Casillo et al., 2020). This “unique” factor, highly associated with chronic inflammatory disease, also plays a role in the cardiovascular system (Maione, 2016). The pathological feature of COVID-19 is that active viral replication activates a variety of immune cells and produces a large number of inflammatory cytokines such as TNF, IL-6, IL-1β, IL-17 and IFN-γ, resulting in cytokine storm (Zhou et al., 2020a; Del Valle et al., 2020). The levels of these cytokines are positively correlated with the severity of the disease [Liu, Y. et al. 2019-novel coronavirus (2019-nCoV) infections trigger an exaggerated cytokine response aggravating lung injury ChinaXiv http://www.chinaxiv.org/abs/202002.00018 (2020)] and lead to lung injury by further aggravating clinical features, and the core mediator of this cytokine storm and its destructive consequences may be TNF (Ablamunits and Lepsy, 2022). At present, in the absence of specific antiviral therapy to neutralize COVID-19 pathogens, controlling cytokine storm may be an effective treatment. Interestingly, serum TNF levels in patients with COVID-19 were significantly higher, which was positively correlated with disease severity and death (Del Valle et al., 2020), and the hospitalization rate of COVID-19 patients treated with TNF inhibitors was lower (Gianfrancesco et al., 2020), suggesting that TNF inhibitors have potential for the treatment of COVID-19. In addition, in the process of SARS-CoV-2 infection, Karki et al. (Karki et al., 2021) found that only the combination of TNF-α and IFN-γ can induce inflammatory cell death. Treatment with neutralizing antibodies against TNF-α and IFN-γ can protect mice from SARS-CoV-2 infection and block the signaling pathway of inflammatory cell death mediated by cytokines. While Xu et al. found an increased expression of ACE2 in the skin of patients with COVID-19, and the results of virus detection suggested that the skin was the potential host of SARS-CoV-2. After treatment with IL-17 antibody, the expression of ACE2 was down-regulated, indicating that IL-17 antibody may reduce the risk of virus infection by reducing the number of cells interacting with SARS-CoV-2 (Xu et al., 2021). Therefore, can some TNF-α inhibitors (adalimumab, infliximab and golimumab) and IL-17 inhibitors (secukinumab and ixekizumab) which can be effectively used in the treatment of RA, AS and other diseases also treat COVID-19 and produce new indications? At present, some drugs have been selected for clinical trials to try to be used as therapeutic drugs for COVID-19 (such as NCT04425538, NCT04593940, NCT04403243andetal). Through the method of NP, the above part of the study analyzed the common action targets and regulatory signaling pathways of four diseases: COVID-19, RA, AS and GA, and discussed IL-17 signaling pathway and TNF signaling pathway, which are closely related to viral infection and inflammatory response, indicating that the regulation of these two signaling pathways commonly used in the treatment of RA, AS and other diseases can also affect the pathogenesis of COVID-19. Corresponding factor inhibitors (TNF-α inhibitors and IL-17 inhibitors) may also play a role in the treatment of COVID-19, achieving the treatment strategy of “new use of old drugs” and “homotherapy for heteropathy”. With the continuous report of the results of clinical trials, the effectiveness of this treatment strategy will be gradually confirmed.

In addition to factor inhibitors, TCM also plays an important role in the prevention and treatment of global COVID-19, such as Lian Hua Qing Wen (Zheng et al., 2020; Shi et al., 2021), which is providing experience and wisdom for the international community to prevent and control the epidemic, highlighting the advantages of TCM. Recently, the scientific research team in China has successfully obtained the national invention patent authorization of CEP, the active ingredient of TCM. The patent specification shows that CEP 10uM inhibits SARS-CoV-2 replication by 15,393 times, showing a strong ability to inhibit viruses. In terms of resistance to COVID-19 research, CEP also belongs to the “new use of old drugs”. CEP tablets have been used clinically for several decades, mainly for leukopenia caused by radiotherapy and chemotherapy in tumor patients (Wu et al., 2001). The beneficial effect of CEP on leukopenia was first noted in 1965 and then confirmed in the early 1980s (Oyaizu et al., 2001). This drug can prevent leukopenia, especially neutropenia, in patients treated with anti-cancer drugs. For the clinical treatment of leukopenia, the current general treatment dose is 20 mg each time, three times a day. The possible adverse reactions during application are mild gastrointestinal discomfort, itchy skin, and local skin and nail bed pigmentation in a few cases. Moreover, a large multicenter clinical study has shown that CEP 30 mg is safe and significantly effective in the treatment of radiation-induced leukopenia, protecting white blood cells from radiation damage and having no effect on red blood cell and platelet counts (Kanamori et al., 2016). Judging from the current progress, the research of CEP still stays in the *in vitro* cell experiment, and whether it is expected to become a therapeutic drug for COVID-19 still needs to go through animal experiments, human clinical trials, market approval and other links. In this study, through the methods of NP and molecular docking, starting from the common action targets of CEP and COVID-19, the enrichment analysis of GO and KEGG was carried out. It was found that CEP interfered the pathogenesis of COVID-19 mainly through the regulation of PI3K-Akt signaling pathway, Relaxin signaling pathway, VEGF signaling pathway and HIF-1 signaling pathway. And the key targets of these important pathways, such as AKT1, PIK3CA, PIK3CD and CEP, all have good binding energy (Figures 5F–H and Table 2), which reveals that CEP treats COVID-19 mainly by acting on these key targets and regulating related pathways. It has been found that SARS-CoV-2 infection can activate PI3K/Akt/mTOR signaling pathway and its downstream targets, resulting in rapid activation of translation mechanisms involved in viral protein synthesis and intracellular phosphorylation (Kindrachuk et al., 2015). In particular, CD147, which is involved in the entry of SARS-CoV-2 into cells, can induce the activation of PI3K/Akt signaling pathway, while the closure of this pathway will inhibit the entry of some viruses (Khezri, 2021). Pulmonary fibrosis is a pathological feature of pulmonary infection in patients with COVID-19. It has been confirmed that Relaxin has the effect of anti-fibrosis and can improve pulmonary symptoms by controlling inflammation and fibrosis (Chow et al., 2012). Simultaneously, Relaxin can also effectively prevent and reverse the ischemia-reperfusion injury in cardiovascular animal model, increase the blood flow of coronary artery and prevent the possible injury of cardiomyocytes caused by COVID-19 (Masini et al., 1996; Bani et al., 1998). In addition, VEGF signaling pathway and HIF-1 signaling pathway are also rich in key targets such as AKT1, PIK3CA and PIK3CD, which may be related to the effect of CEP on COVID-19. In SARS-CoV-2 infection, the increased level of VEGF leads to high permeability, edema and tissue injury, which triggers the pathogenesis of acute lung injury in patients with COVID-19 (Teuwen et al., 2020; Wu and McGoogan, 2020). On the other hand, VEGF signaling pathway increases the level of angiotensin II (Ang II) to promote inflammation, whereas Ang II can increase vascular endothelial growth factor (VEGF), thus forming a vicious circle in the release of inflammatory cytokines (Shang et al., 2019). Finally, it causes the immune cells in the body to secrete a large number of inflammatory factors, form a cytokine storm, attack the immune organs in the human body, seriously affect the respiratory and circulatory systems, and even lead to the death of patients. The inflammatory response of lung can lead to respiratory distress and hypoxia. HIF-1 signaling pathway participates in the regulation of hypoxia in the human body and can activate the transcription of multiple genes under hypoxia conditions. Hypoxia-inducible factor-1 (HIF-1), as one of the hypoxia signal transcription factors (Zhang et al., 2017; Tian et al., 2021), regulates macrophages and T cells by regulating the expression of metabolism-related genes, thereby promoting inflammatory response (Ferraro et al., 2021). Therefore, CEP may play an anti-hypoxia effect by regulating HIF-1 signaling pathway and alleviate the clinical hypoxia symptoms of patients with severe COVID-19. Finally, CEP was docked with ACE2, a key protein in the pathogenesis of COVID-19. It is reported that ACE2 is the receptor protein (Harrison et al., 2020) of respiratory tract, alveolar and vascular endothelial cells. SARS-CoV-2 induces the internalization and exfoliation of ACE2 through the binding of S-protein to ACE2, and then invades the body, leading to the occurrence and development of acute respiratory distress syndrome (Li et al., 2005; Zhou P. et al., 2020). The combination of SARS-CoV-2 and ACE2 is the main cause of COVID-19, and SARS-CoV-2 can only enter cells that can express ACE2. Therefore, ACE2 plays an important role in SARS-CoV-2 infection (Zhang et al., 2020). The results show that CEP and ACE2 have strong binding activity, reaching -12.44 kcal/mol (Figure 5I; Table 2), which provides some data reference and support for deeply analyzing the blood components of CEP to prevent COVID-19. In this part, we explore the action mechanism of CEP through the methods of NP and molecular docking technology, and find that CEP acts on the key targets such as AKT1, PIK3CA, PIK3CD, and ACE2 through PI3K-Akt signaling pathway, Relaxin signaling pathway, VEGF signaling pathway and HIF-1 signaling pathway to achieve the purpose of anti-COVID-19, which lays a foundation for the further study of CEP and drug screening for prevention and treatment of COVID-19.

## Conclusion

To sum up, we systematically analyzed the common action targets and signaling pathways of four diseases, including COVID-19, RA, AS and GA, based on the NP and the theory of “homotherapy for heteropathy”, and revealed some commonness in their pathogenesis. It is explained theoretically that the “new use of old drugs” of TNF and IL-17 inhibitors increases the possibility of treating COVID-19 as a new indication. Although there are nearly 100 clinical trials related to COVID-19 at home and abroad, up to now, there are no specific therapeutic drugs, and the research and development of vaccine has its lag. Therefore, this study focused on the relatively safe and commonly used TCM extract CEP, and analyzed the therapeutic target, signal transduction pathway, possible mechanism and molecular docking pathway of CEP in the treatment of COVID-19. The purpose of this study is to provide a reference basis for the clinical application of CEP and to further explore its anti-COVID-19 mechanism, but in view of the limitations of the analysis method, the results need to be further verified.

## Data Availability

Publicly available datasets were analyzed in this study. This data can be found here: https://www.genecards.org/http://www, https://pubchem.ncbi.nlm.nih.gov/, http://www.swisstargetprediction.ch/, https://omim.org/, https://db.idrblab.org/ttd/, https://www.pharmgkb.org/.

## Abbreviations

ACE2, angiotensin-converting enzyme 2; ALB, albumin; Ang II, angiotensin II; AS, ankylosing spondylitis; AKT1, AKT Serine/Threonine Kinase 1; CEP, Cepharanthine; COVID-19, Corona Virus Disease 2019; GA, gouty arthritis; GO, gene ontology; HIF-1, hypoxia-inducible factor-1; IFN, interferon; IL, interleukin; JAK, Janus Kinase; KEGG, kyoto encyclopedia of genes and genomes; NP, network pharmacology; OMIM, Online Mendelian Inheritance in Man; PAMP, pathogen-associated molecular pattern; PIK3, phosphatidylinositol 3-kinase; PPI, protein-protein interactions; RA, rheumatoid arthritis; SARS-CoV-2, severe acute respiratory syndrome coronavirus 2; TCM, traditional Chinese medicine; TTD, Therapeutic Target Database; TCMSP, traditional Chinese medicine systems pharmacology database and analysis platform; TLR4, Toll-like receptor 4; VEGF, vascular endothelial growth factor.
